# Use of Web-Based Health Services in Individuals With and Without Symptoms of Hypochondria: Survey Study

**DOI:** 10.2196/10980

**Published:** 2019-06-10

**Authors:** Christiane Eichenberg, Markus Schott

**Affiliations:** 1 Sigmund Freud Privat Universität Wien Vienna Austria

**Keywords:** hypochondria, eHealth, anxiety, survey

## Abstract

**Background:**

An increasing number of people consult physicians because of distressing information found online. Cyberchondria refers to the phenomenon of health anxiety because of online health information.

**Objective:**

This study aimed to examine online health research of individuals with and without symptoms of hypochondria and their impact on health anxiety as well as behavior.

**Methods:**

An online survey was conducted. Demographic data, health-related internet use, and general health behavior were assessed. The illness attitude scale was used to record symptoms of hypochondria.

**Results:**

The final sample consisted of N=471 participants. More than 40% (188/471) of participants showed at least some symptoms of hypochondria. Participants with symptoms of hypochondria used the internet more frequently for health-related purposes and also frequented more online services than individuals without symptoms. Most online health services were rated as more reliable by individuals with symptoms of hypochondria. Changes to behavior such as doctor hopping or ordering nonprescribed medicine online were considered more likely by individuals with symptoms of hypochondria.

**Conclusions:**

Results show that individuals with symptoms of hypochondria do not turn to online research as a result of lacking alternatives but rather consult health services on- as well as offline.

## Introduction

### Background

Currently, the internet provides an enormous amount of information about a range of health topics. According to a representative study with N=2411 participants [1], 63.5% of German internet users rely on the internet as a guide concerning health-related issues. In addition to traditional media such as television, radio, and print, the internet has become an important source of information related to health issues. Information found online does not affect personal health behavior as much as a doctor’s or family member’s recommendation; however, their importance is comparable with the influence of a pharmacist [1]. According to a US representative telephone survey [2], 86% of online health users reported that online research had successfully met their information needs and also regarded the information found as reliable.

Online health research may lead to positive and preventative activities such as exercise, healthier eating habits, improved adherence to medication, and empowered health decisions [3]. However, these possibilities present a problem when used as diagnostic tools by laypersons [4]. As the internet provides access to a large body of information, it is a crude means of self-diagnosis. For example, multiple medical possibilities are typically presented in response to internet searches of medical concerns [5]. Therefore, searching for medical information on the internet has the potential to lead to even greater levels of uncertainty and consequently exacerbate health anxiety for vulnerable individuals. This might aggravate already existing worries about being sick with a certain disease for patients suffering from hypochondria.

### Definition

Hypochondria is a debilitating disorder that occurs in about 1 to 5% of people once in a lifetime [6]. The Diagnostic and Statistical Manual of Mental Disorders (DSM), 4th edition (Text Revision) defines this disorder as a somatoform disorder [7]. The newly published DSM 5^th^ edition replaces the diagnosis of hypochondria with the diagnoses of somatic symptom disorder and illness anxiety disorder [8]. Hypochondria refers to a persistent preoccupation with, or fears about the possibility of having or developing 1 or more serious progressive or life-threatening diseases. The preoccupation is associated with a hypervigilance to and catastrophic misinterpretation of bodily signs or symptoms including normal sensations and is accompanied by avoidance or repetitive behaviors. Minor symptoms are thus interpreted as evidence of suspected diseases, and concerns persist despite reassurance by physicians [7,9]. Disease conviction can be maintained by repetitive symptom- checking or reassurance seeking including activities on the internet: retrieving medical information or entering self-observed symptoms into online diagnostic systems. In addition, sharing information online with other affected people may be an attempt to get rid of unpleasant thoughts and feelings concerning their own state of health.

This more recent phenomenon has been referred to as cyberchondria [10]. Cyberchondria has been defined as an excessive search on the internet for health-related information, which is driven by an underlying need to ease anxiety, but subsequently results in symptoms worsening [11]. It is a form of reassurance-seeking behavior. Instead of obtaining support via online interactions with similarly worried individuals, those with cyberchondria experience their health anxiety as exacerbated, mainly because of new pathologies found online that might trigger new worries [2]. Although current research already declared the phenomenon of cyberchondria as a distinct clinical disorder [10], the prevailing view is that cyberchondria is part of hypochondria/health anxiety. Unfortunately, little research has been conducted in this area. There have only been few elaborate studies on internet use and health fears to date.

### Available Research

A survey of over 500 respondents by White and Horvitz [10] found that more than 60% of participants repeatedly searched for a specific health concern in multiple online sessions. In a further analysis of thousands of online interaction logs, it was found that over an 11-month period, 13.5% of study participants entered the exact same health-related terms into a search engine on more than 1 occasion [10]. Starcevic and Berle [11] suggested that these excessive searches only serve to reinforce a person’s original anxiety. Over a third of the sample (n=198, 38.4%) said they experienced an increase in anxiety following an online search for health information. The majority (n=365, 70.7%) of participants in this study believed that the fear was caused by the content of websites visited as part of their research efforts; that is, navigation escalation. A total of 7 out of 10 respondents were still researching serious illnesses for weeks and months after a conducted search, suggesting that these escalated search sessions had a lasting impact.

Aside from causing unwarranted levels of worry and distress, there may also be economic costs to cyberchondria [12]. Even though no studies have examined the costs directly associated to online health searches to this date, there is evidence that those who are especially health anxious constitute a considerable economic burden to society. For example, health care costs and losses in productivity attributable to medically unexplained symptoms account for an estimated £3 billion in 2008 in the United Kingdom alone [13]. Cyberchondria may be likely to be responsible for a significant proportion of this amount. In an online study (N=240), Eastin and Guinsler [14] found a moderating effect of health anxiety on the link between health-related online research and the use of health care. Results highlighted that with increasing health anxiety, the association between the extent of online information search and appointments with physicians became stronger. Thus, health anxiety seems to be an important determinant of how online medical research affects the decision to visit a doctor. Furthermore, an analysis of anonymous search logs showed that those who frequently searched for health information online ended their search sessions with queries about local health care services [15]. In contrast, research has also suggested that cyberchondria can lead to deterioration within the doctor-patient relationship [16], which may lead to further health care costs (eg, visits to multiple doctors). Therefore, it is important to better understand this disorder to inform new strategies to minimize its negative consequences.

### Objectives

This study aimed to examine usage patterns of online health research and their impact on health anxiety and behavior. It was hypothesized that individuals with symptoms of hypochondria would use different online health services more often than individuals without symptoms of hypochondria. Furthermore, it was hypothesized that individuals with symptoms of hypochondria would evaluate these services with regard to information quality better than individuals without symptoms of hypochondria*.* We also investigated how participants react to health-related online research and how they rated their potential to alleviate anxiety.

## Methods

### Study Design

The study was carried out as a Web-based survey. To generate a sample as heterogeneous as possible, the questionnaire was distributed to more than 180 German-speaking health forums, ranging from very specific health-related forums (eg, online self-help for alcoholics) to more general ones. Information about the purpose and the course of the study alongside an invitation link to the online questionnaire were published in the forums. The data collection period lasted 4 weeks. Completing the questionnaire took about 10 to 15 min.

Ethical approval was obtained from the Sigmund Freud University Vienna ethics committee (QBABQXGPA@ APWF87082).

Statistical analyses were conducted by using ASW Statistics for Windows, Version 18.0 by SPSS Inc.

### Material

The online questionnaire consisted of 2 parts: Part I was self-constructed and included demographic data (5 items), health-related internet use (11 items), and general health behavior (7 items). Part II was standardized and assessed existing symptoms of hypochondria.

A pretest was carried out in a specially selected health forum. The N=17 returns were analyzed, and the instrument was revised regarding its practicability, comprehensibility, and completeness of item formulation.

#### Demographic Data, Internet Use, and Health Behavior

In addition to age, gender, and nationality of participants, the highest level of education and subjects’ current occupational status were assessed.

To acquire information about the usage of health-related online services, participants were asked to report their level of internet literacy, the frequency of internet use, as well as internet services used for health-related research. It was also recorded how subjects perceived the quality of various online health services and whether participants experienced effects of different online health services on one’s own health anxiety.

Study participants indicated the availability of adequate medical care in the immediate vicinity on a 5-point Likert scale (“very good” to “deficient”), the frequency of doctor visits, and number of different doctors visited within the last year.

#### Illness Attitude Scale

The Illness Attitude Scale (IAS) was used to record health anxiety. This instrument consists of 2 scales (1st disease anxiety, 2nd disease behavior) with 29 items on various aspects of hypochondria to be answered on a 5-step scale. A total of 9 scales are formed from 3 questions each. In addition, 2 additional questions are included. The IAS has 9 subscales: (1) worry about illness, (2) concerns about pain, (3), health habits, (4) hypochondriacal beliefs, (5) thanatophobia (fear of death), (6) disease phobia, (7) bodily preoccupations, (8) treatment experience, and (9) effects of symptoms. Each item is rated on a 0 to 4 Likert scale, and 27 of the 29 items are used in the total score, which ranges from 0 to 108. Findings show that a cut-off of 47 points yields a sensitivity of 96% and specificity of 95%. The Illness Attitude Scale has a retest-reliability between 0.89 and 0.93 and a Cronbach’s Alpha of α = 0.90 [17].

## Results

### Sample

The final sample consisted of N=471 participants recruited from various health forums on the internet, of which 397 (84.3%) were female. Participants were aged between 14 and 84 years (mean 40.0, SD 13.25). Most participants were in a relationship or married (310/471, 65.8%). At the time of the survey, more than half of participants (273/471, 57.9%) had finished school with at least a high school diploma.

#### Illness Attitude Scale

Results indicated that more than a quarter of participants showed at least some symptoms of hypochondria (70/471, 14.9%) or could even be diagnosed with hypochondria (120/471, 25.5%). To ease statistical analyses and to create more balanced group sizes, these 2 categories were merged into 1 (190/471, 40.4%).

### Comparison Between Participants With and Without Hypochondria

#### Internet Use

Participants used a wide variety of services on the internet on a fairly regular basis (Table 1). Consequently, it is not surprising that a majority of subjects rated their own internet literacy as “advanced” (334/471, 70.9%) or even “expert” (67/471, 14.3%).

Half of the sample reported that they had used the internet more than 10 times in the past year to search for acute (215/471, 45.7%) or chronic symptoms (217/471, 46.2%). As expected, results showed that individuals with symptoms of hypochondria had been searching for their own acute (χ^2^_4_=28.82; n=400; *P*<.001; phi=0.27) and chronic symptoms χ^2^_4_=28.167; n=400; *P*<.001; phi=0.27) significantly more often than those with low health anxiety. For example, 76% (144/190) of individuals with symptoms of hypochondria searched for symptoms more than 10 times on the internet last year compared with only 36.3% (n=101/281) of individuals without symptoms of hypochondria.

**Figure 1:**
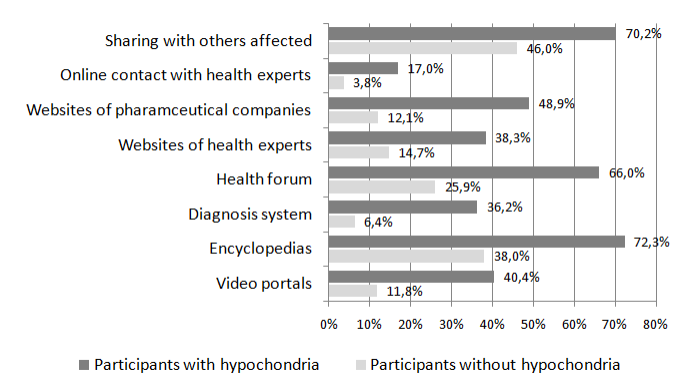
Weekly or more often usage of different online services for individuals with and without symptoms of hypochondria.

Individuals with symptoms of hypochondria also differed significantly in the types of online health services used from individuals without symptoms of hypochondria (all differences *P*<.001; see Figure 1). In particular, health services to share with others affected and health forums were used several times a day by 35% (66/281) and 50% (95/281) of individuals with symptoms of hypochondria, respectively, whereas only 12.6% (35/281) of individuals without symptoms of hypochondria used these services on a daily basis.

**Table 1:** Intensity of use of various internet services (N=470).

Reason to use	Frequency			
	At least once a day, n (%)	At least once a week, n (%)	At least once a month, n (%)	Never, n (%)
Professional	198 (42.2)	92 (19.6)	63 (13.4)	115 (24.5)
Entertainment	282 (60.1)	119 (25.3)	23 (4.9)	22 (4.7)
Social media	295 (62.8)	130 (27.7)	17 (3.6)	28 (6)
Information	323 (68.7)	133 (28.5)	10 (2.2)	3 (0.6)

Similarly, individuals with symptoms of hypochondria used a greater variety of services than individuals without symptoms of hypochondria (*t*_358_=–4.06; *P*<.001; *d*=0.65). Individuals with symptoms of hypochondria consulted on average mean 5.72 (SD 1.79) various health services on the internet, whereas individuals without these symptoms only visited mean 4.51 (SD 1.93) services.

#### Evaluation of Online Information Quality

In general, study participants’ appraisal of online health information was critical. The health service with the highest rating was only considered to be “rather reliable.” The quality of online help by experts (mean 1.97, SD 0.81) was rated highest. However, exchange with medical laypersons (mean 1.77, SD 0.81) was considered to be almost as reliable. In contrast, information from pharmaceutical companies (mean 1.26, SD 0.86) was assessed more critically, similar to online diagnostic systems (mean 0.82, SD 0.79) and information on video portals (mean 0.53, SD 0.71).

Unsurprisingly, most online health services were rated as significantly more reliable by individuals with symptoms of hypochondria than by individuals without symptoms of hypochondria (see Table 2).

**Table 2:** Evaluation online information quality.

Online services		Mean (SD)	*t* (*df*)	Cohen *d*	*P* value
**Online contact with experts**		—^a^	–0.53 (213)	0.10	.59
	Symptoms of hypochondria	2.04 (0.88)	—	—	—
	No symptoms of hypochondria	1.95 (0.80)	—	—	—
**Sharing with others affected**		—	–2.26 (363)	0.36	.02
	Symptoms of hypochondria	2.00 (0.78)	—	—	—
	No symptoms of hypochondria	1.71 (0.82)	—	—	—
**Websites of pharmaceutical companies**		—	–3.18 (294)	0.54	.002
	Symptoms of hypochondria	1.67 (0.86)	—	—	—
	No symptoms of hypochondria	1.21 (0.84)	—	—	—
**Websites of health experts**		—	–1.94 (276)	0.19	.05
	Symptoms of hypochondria	2.00 (0.83)	—	—	—
	No symptoms of hypochondria	1.74 (0.74)	—	—	—
**Professional health portal**		—	–0.67 (329)	0.12	.50
	Symptoms of hypochondria	1.93 (0.67)	—	—	—
	No symptoms of hypochondria	1.85 (0.69)	—	—	—
**Online diagnosis system**		—	–2.88 (42)	1.58	.01
	Symptoms of hypochondria	2.00 (0.83)	—	—	—
	No symptoms of hypochondria	0.76 (0.74)	—	—	—
**Online encyclopedias**		—	–2.21 (60)	0.33	.03
	Symptoms of hypochondria	1.93 (72)	—	—	—
	No symptoms of hypochondria	1.68 (0.80)	—	—	—
**Video portals**		—	–3.84 (241)	0.64	< .001
	Symptoms of hypochondria	1.00 (0.87)	—	—	—
	No symptoms of hypochondria	0.50 (0.67)	—	—	—

^a^Not applicable.

#### Evaluation of the Potential of Researched Information to Alleviate Anxiety

Overall, results showed that sharing with others affected (mean 1.94, SD 0.83) and online contact with experts (mean 1.93, SD 0.83) were able to alleviate anxiety. However, online diagnosis systems (mean 1.10, SD 0.77) and video platforms (mean 0.98, SD 0.83) seemed to cause the opposite.

However, individuals with symptoms of hypochondria did not rate health services significantly different with regard to their potential to alleviate anxiety in comparison with individuals without symptoms of hypochondria (see Table 3).

**Table 3:** Potential of researched information to alleviate anxiety.

Online services		Mean (SD)	*t* (*df*)	Cohen *d*	*P* value
**Online contact with experts**		—^a^	1.31 (25.76)	0.35	.20
	Symptoms of hypochondria	1.68 (1.08)	—	—	—
	No symptoms of hypochondria	2.0 (0.69)	—	—	—
**Sharing with others affected**		—	0.15 (254)	0.01	.88
	Symptoms of hypochondria	1.95 (0.82)	—	—	—
	No symptoms of hypochondria	1.93 (0.93)	—	—	—
**Websites of pharmaceutical companies**		—	–2.09 (167)	0.37	.04
	Symptoms of hypochondria	1.64 (0.77)	—	—	—
	No symptoms of hypochondria	1.37 (0.67)	—	—	—
**Websites of health experts**		—	–0.17 (37)	0.04	.86
	Symptoms of hypochondria	1.77 (0.95)	—	—	—
	No symptoms of hypochondria	1.74 (0.65)	—	—	—
**Professional health portal**		—	0.72 (213)	0.02	.47
	Symptoms of hypochondria	1.61 (0.82)	—	—	—
	No symptoms of hypochondria	1.70 (0.72)	—	—	—
**Online diagnosis system**		—	–0.97 (45)	0.22	.33
	Symptoms of hypochondria	1.19 (0.93)	—	—	—
	No symptoms of hypochondria	1.01 (0.72)	—	—	—
**Online encyclopedias**		—	1.59 (250)	0.27	.11
	Symptoms of hypochondria	1.40 (0.80)	—	—	—
	No symptoms of hypochondria	1.60 (0.71)	—	—	—
**Video portals**		—	0.44 (92)	0.09	.66
	Symptoms of hypochondria	0.96 (0.86)	—	—	—
	No symptoms of hypochondria	1.04 (0.86)	—	—	—

^a^Not applicable.

#### Health Behavior

Respondents seemed to be satisfied with general health care provision. Most participants were satisfied with the availability of medical services in their direct local area (mean 2.11, SD 1.07). In the last year, 80.2% (376/471) of the total sample consulted a doctor. Participants visited on average 3.55 (SD 3.01) different doctors 11.65 times (SD 16.0) in the last year.

Participants with symptoms of hypochondria considered all suggested behaviors to be more likely as a reaction to the use of online health services as participants without symptoms of hypochondria. Individuals with symptoms of hypochondria reported having visited a doctor significantly more often after health-related online searches (*P*<.001) compared with individuals without symptoms of hypochondria. Individuals with symptoms of hypochondria were also more likely to include ordering medication on the internet (*P*<.001). Furthermore, additional searches for health information as a response to the use of internet health services were considered much more probable by individuals with symptoms of hypochondria (mean 3.67, SD 1.27) than by individuals without symptoms of hypochondria (mean 2.43, SD 1.24; *P*<.001).

## Discussion

### Principal Findings

Cyberchondria is a form of health anxiety characterized by excessive online health research. As there have only been few elaborate studies on internet use and health fears to date, this study aimed to examine online health research of individuals with and without symptoms of hypochondria and their impact on health anxiety as well as behavior.

In the current sample of German online health service users, the portion of individuals with symptoms of hypochondria was as high as one-fourth (25.5%). In contrast, the prevalence of hypochondria in the general population is estimated at 6.7% [18]. This could be because of our recruitment methods of advertising primarily on health information websites, as persons who tend to be anxious regarding health issues consult these services more frequently [19]. Results showed that health-related internet services were used more frequently by individuals with symptoms of hypochondria. In addition, these individuals also used a greater variety of services. In line with available research, seeking online health information had a detrimental impact on those with health anxiety [19]. Results showed that information researched online had a greater impact on health behavior in individuals with symptoms of hypochondria. Therefore, despite the established benefits of online health services such as the empowerment of patients [20], searching online health sites may carry a risk of aggravating health anxiety and lead to dysfunctional health behaviors such as doctor hopping, ordering nonprescribed medicine online, or intensifying their online research. However, studies show that the quality of information in health websites is often questionable [21]. As a consequence, individuals may have difficulties differentiating between reliable websites and websites with less reliable information. In this study, there were no significant differences in the assessment of the quality of online health services between individuals with and without symptoms of hypochondria. Similar to previous research, participants in this study perceived online health information to be somewhat reliable [22,23]. Thus, it is important to minimize misleading information on the internet. A possible solution to differentiate between websites of poor and high quality may be to label high-quality services with appropriate seals of approval and make these websites readily recognizable.

Patients suffering from hypochondriasis as well as people without this psychopathology agree that sharing worries with others—similar to an online contact with an expert—is able to alleviate health anxiety. In this sense, personal relationships, whether it is being part of a community or with a competent person, have the highest impact on gaining a feeling of security. Taking into account this relationship, an increasing intensity of medical internet research is accompanied with a greater use of medical care provision. Consequently, it is not surprising that individuals with symptoms of hypochondria reported consulting a medical professional as a consequence of online health research more often than individuals without symptoms of hypochondria. This shows that individuals with symptoms of hypochondria do not turn to online research as a result of lacking alternatives but rather consult them on- as well as offline. In conclusion, use of health-related websites may act as a catalyst for symptoms of hypochondria, reinforcing symptoms or increasing use of medical services.

### Limitations and Future Research

There are some methodological limitations to this study. First, possible self-selection processes should be noted. Online surveys are predisposed for an inherent selection bias by being limited to those with access to computers and internet resources. It is possible that the number of individuals with symptoms of hypochondria in the study sample might be overestimated. Considering the significant higher amount of participants with hypochondria in this sample compared with data for the general population, this needs to be kept in mind. This might result from the fact that individuals with symptoms of hypochondria visit online health services more frequently than individuals without these symptoms [19]. In addition, to balance group sizes, participants who showed at least some symptoms of hypochondria were combined with those that could be diagnosed with hypochondria. Female participants contributed disproportionately to the respondent dataset. However, this gender bias in online surveys has been frequently observed in the literature [24]. Therefore, results cannot be considered as representative of all internet users.

To increase the range of results, a representative offline study as well as supplementary screening by the attending physician would be desirable. Thereby, self-assessment data could be validated and extended. In addition, it is necessary to investigate the association between personality traits, health-related online content, and cognitive as well as emotional processes. Thereby, studies may shift light on the role of moderating factors on the association between health anxiety and internet usage.

## Abbreviations

DSM: Diagnostic and Statistical Manual of Mental Disorders
IAS: Illness Attitude Scale

## References

[1] Eichenberg C, Wolters C, Brähler E (2013). The internet as a mental health advisor in Germany--results of a national survey. PLoS One.

[2] Starcevic V, Aboujaoude E (2015). Cyberchondria, cyberbullying, cybersuicide, cybersex:. World Psychiatry.

[3] Huberty J, Dinkel D, Beets MW, Coleman J (2013). Describing the use of the internet for health, physical activity, and nutrition information in pregnant women. Matern Child Health J.

[4] Berle D (2015). Cyberchondria. Mental Health in the Digital Age: Grave Dangers, Great Promise.

[5] Fergus TA (2013). Cyberchondria and intolerance of uncertainty: examining when individuals experience health anxiety in response to Internet searches for medical information. Cyberpsychol Behav Soc Netw.

[6] Leigh H, Streltzer J (2015). Handbook of Consultation-Liaison Psychiatry.

[7] (2000). Diagnostic and Statistical Manual of Mental Disorders: DSM-IV-TR.

[8] (2013). Diagnostic and Statistical Manual of Mental Disorders: DSM-V.

[9] van den Heuvel OA, Veale D, Stein DJ (2014). Hypochondriasis: considerations for ICD-11. Braz J Psychiatry.

[10] White RW, Horvitz E (2009). Cyberchondria: studies of the escalation of medical concerns in Web search. ACM Trans Inf Syst.

[11] Starcevic V, Berle D (2013). Cyberchondria: towards a better understanding of excessive health-related Internet use. Expert Rev Neurother.

[12] McElroy E, Shevlin M (2014). The development and initial validation of the cyberchondria severity scale (CSS). J Anxiety Disord.

[13] Bermingham S, Cohen A, Hague J, Parsonage M (2010). The cost of somatisation among the working-age population in England for the year 2008-2009. Ment Health Fam Med.

[14] Eastin MS, Guinsler NM (2006). Worried and wired: effects of health anxiety on information-seeking and health care utilization behaviors. Cyberpsychol Behav.

[15] White R, Horvitz E (2010). Web to world: predicting transitions from self-diagnosis to the pursuit of local medical assistance in web search. AMIA Annu Symp Proc.

[16] Keller GL, Padala PR, Petty F (2008). Clinical pearls to manage cyberchondriacs. Prim Care Companion J Clin Psychiatry.

[17] Gmelch M, Reinecker H (2006). [International scales for hypochondria. German-language adaptation of the Whiteley Index (WI) and the Illness Attitude Scales (IAS)]. Z Klin Psychol Psychopathol Psychother.

[18] Bleichhardt G, Hiller W (2007). Hypochondriasis and health anxiety in the German population. Br J Health Psychol.

[19] Muse K, McManus F, Leung C, Meghreblian B, Williams JM (2012). Cyberchondriasis: fact or fiction? A preliminary examination of the relationship between health anxiety and searching for health information on the Internet. J Anxiety Disord.

[20] van Uden-Kraan C, Drossaert C, Taal E, Seydel E, van de Laar M (2009). Participation in online patient support groups endorses patients' empowerment. Patient Educ Couns.

[21] Eichenberg C, Blokus G, Malberg D (2013). [Evidence-based patient information on the web - a study on the quality of post traumatic stress disease sites]. Z Klin Psychol Psychopathol Psychother.

[22] Murray E, Lo B, Pollack L, Donelan K, Catania J, Lee K, Zapert K, Turner R (2003). The impact of health information on the internet on health care and the physician-patient relationship: national US survey among 1.050 US physicians. J Med Internet Res.

[23] Peterson MW, Fretz PC (2003). Patient use of the internet for information in a lung cancer clinic. Chest.

[24] Jackob N (2009). [Social Research On the Internet: Methodology and Practice of the Online Survey].

